# Spatiotemporal Dual-Channel Interpretable Hybrid Neural Network for HD-sEMG-Based Gesture Recognition

**DOI:** 10.3390/s26144602

**Published:** 2026-07-20

**Authors:** Zhefei Cai, Su Liu, Xinyue Li, Michael Houston, Yingle Fan, Yingchun Zhang

**Affiliations:** 1 College of Information Engineering, China Jiliang University, Hangzhou 310018, China; zfcai@cjlu.edu.cn; 2 Department of Biomedical Engineering, University of Miami, Coral Gables, FL 33146, USA; suliu@med.miami.edu (S.L.); xxl1189@miami.edu (X.L.); mxh1983@miami.edu (M.H.); 3 School of Automation, Hangzhou Dianzi University, Hangzhou 310018, China; 4 Department of Neurology, University of Miami Miller School of Medicine, Miami, FL 33136, USA; 5 Desai Sethi Urology Institute, University of Miami Miller School of Medicine, Miami, FL 33136, USA; 6 Miami Project to Cure Paralysis, University of Miami, Miami, FL 33136, USA

**Keywords:** interpretable neural network, graph attention network, HD-sEMG classification, SHAP value

## Abstract

**Highlights:**

- Developed a spatiotemporal dual-channel interpretable hybrid neural network (STDC-Net) that integrates complementary feature-based and spatial representations of high-density surface EMG (HD-sEMG) signals. The feature channel extracts time- and frequency-domain features with SHAP-based importance ranking to guide learning, while the spatial channel processes raw signals using temporal convolutions and graph attention to model spatiotemporal dynamics and inter-muscle connectivity, enhancing interpretability and performance.
- Demonstrated state-of-the-art performance for HD-sEMG gesture recognition. The developed STDC-Net achieved 99.8% accuracy with a 150 ms sliding window, exceeding real-time implementation requirements for intra-subject tasks. It reached an accuracy of 97.33 ± 2.53% after fine-tuning for inter-subject tasks, outperforming the SOTA methods.

**Abstract:**

Accurate gesture recognition is crucial for precision control of upper limb prostheses. High-density surface electromyography (HD-sEMG) enhances spatial resolution and information richness of human gesture representation, thus improving myoelectric control of bionic limbs. Recently, deep learning has been increasingly applied to HD-sEMG to enhance gesture recognition performance. However, the black-box nature of neural networks limits their interpretability and model optimization, hindering their practical application. In this paper, we developed a spatiotemporal dual-channel interpretable hybrid neural network (STDC-Net), and validated it using the Capgmyo DB-a dataset. STDC-Net uses Feature Channels and Spatial Channels to process the feature and spatial information of sEMG signals respectively for increased interpretability. SHapley Additive exPlanations (SHAP) values are used to rank the feature importance, aiding feature filtering and reducing the impact of irrelevant features. Graph attention layers are used to calculate the connections between each Spatial Channel, illustrating the relationships between channels. Our results demonstrated the superior performance of our STDC-Net compared to state-of-the-art (SOTA) methods. STDC-Net achieved 99.8% accuracy with a 150 ms sliding window, exceeding real-time implementation requirements for intra-subject tasks. It reached an accuracy of 97.33 ± 2.53% after fine-tuning for inter-subject tasks, outperforming the SOTA methods. Importantly, the SHAP value maps and the channel connection maps enhance the interpretability of the neural networks by offering detailed insights into the contribution of input features and parameter interactions of the network. These findings suggest STDC-Net holds significant promise for real-time prosthetic control.

## 1. Introduction

Limb motor dysfunction affects a growing number of individuals following traumatic brain or spinal cord injury. It is essential to improve or restore the patient’s activities of daily living through rehabilitation [1]. However, the extraordinary degrees of freedom involved in inter-muscular coordination of human upper limbs makes it challenging to accurately identify fine motor control strategies such as those required for gesture control. This knowledge gap makes precise gesture recognition a persistent challenge in the field, which, otherwise, could significantly enhance the performance of hand prostheses. High-density surface electromyography (HD-sEMG) signals capture myoelectric activities with significantly higher spatiotemporal resolution compared to traditional sEMG [2,3]. This allows HD-sEMG to more precisely characterize the generation and propagation of motor unit action potentials which carry rich information related to the descending neural drive to muscles [4,5,6], thereby enabling noninvasive interfacing of human motor intention with robotic actions [7].

Gesture recognition methods generally fall into two categories: conventional methods and deep learning-based methods. Conventional methods rely on temporal/spectral feature extraction, followed by the application of classification methods [8]. While these methods have a wide range of applications and show promise, they are limited by the need for manual feature extraction which may not fully utilize the physiological context of the sEMG during tasks. Deep learning-based methods, in contrast, utilize end-to-end recognition patterns, combining feature extraction and recognition to automatically extract relevant features for classification [9]. A large amount of Convolutional Neural Network-based (CNN-based) classifiers have since emerged [10,11,12,13], surpassing various feature-engineering techniques in accuracy [14,15]. While CNN-based methods are capable of handling time domain and frequency domain effectively, they overlook the spatiotemporal relevance, that is, the combination of spatial and temporal features of muscle signals. Both the special distribution of electrodes and time-varying characteristics of EMG signals should be considered in accurate hand gesture classification. Muscle groups responsible for gesture control are inherently located adjacent to one another in the forearms, hands and fingers. To address spatial dependencies in muscle activity, geometric deep learning methods, such as graph convolutional networks (GCNs) [16] and graph attention networks (GATs) [17], have been introduced. GCNs achieved an impressive accuracy of 91.25% when initially employed in HD-sEMG-based gesture recognition [18], while CNN-GCN hybrid networks have further enhanced the detection performance ever since [19,20]. While GCNs provide valuable insights into the representation of non-Euclidean data, the heuristic distance-based approach of GCNs may omit information of connectivity strengths between muscle groups. Representative hybrid approaches include STGCN-GR [21], which constructs muscle networks based on functional connectivity between channels and applies spatiotemporal graph convolution to capture both spatial topology and temporal dependencies. CNN-MSTGCN [22] integrates a CNN feature extraction front-end with a multi-view spatial–temporal graph convolutional network, and DGTG-wGIN [23] proposes a dynamic graph topology generation mechanism that adaptively establishes graph structure by learning nonlinear mappings between CNN-extracted node features and weighted adjacency matrices. Despite their success, these methods share two common limitations: (1) predominantly relying on either raw signals or simple handcrafted features as inputs, without systematically exploiting the complementary strengths of both; (2) offering limited interpretability regarding which input features or channel relationships are most critical for the network’s decision, hindering model transparency and clinical trust.

However, deep learning models often rely on unexplainable, black-box structures that makes their operation mode largely unclear, limiting optimization and real-world applicability [24]. Accuracy alone is no longer sufficient for model evaluation, as interpretable deep learning is rapidly becoming an emerging hotspot [25]. Interpretable machine learning approaches generally fall into post hoc interpretability and ante hoc interpretability. Post hoc interpretability refers to the interpretability analysis of the trained model, which is suitable for classification problems [26], while the ante hoc interpretability defines models with definite physical meaning [27]. Recent studies in the field of gesture recognition have focused on improving both accuracy and real-time requirements of interpretable network models to decompose the network model and analyze feature weight information. Massa et al. [28] exploited an explainable AI algorithm (XAI) to refine graph topology, improving the recognition accuracy while reducing computational cost. Gozzi et al. [29] proposed an XAI model for a myo-controlled prosthesis to understand the outcome with respect to physiological processes, which evaluates the contribution of each feature to the prediction. This XAI model recognizes hand gestures by mapping and fusing high-amplitude activity of synergic muscles. Lee et al. [30] proposed a graph structure network capturing spatiotemporal features based on the spatial proximity of distributed EMG sensors, offering insights into muscular activation patterns. While interpretable deep learning has shown promising potential, existing solutions fall short in integrating temporal, spectral, and spatial information derived from HD-sEMG in an explainable manner.

In this study, we propose a spatiotemporal dual-channel interpretable hybrid neural network (STDC-Net) to address the aforementioned limitations. The primary novelty of STDC-Net lies in its dual-channel architecture that synergistically combines handcrafted feature-based processing with raw signal-based spatiotemporal modeling, a design fundamentally different from existing single-pathway CNN-GNN approaches. Specifically, the Feature Channel extracts time- and frequency-domain features, with SHAP-based importance ranking providing explicit interpretability of feature contributions. Concurrently, the Spatial Channel processes raw HD-sEMG signals through temporal convolutions and graph attention layers to capture topological relationships and temporal dependencies, with channel connection maps revealing muscle synergies. To the best of our knowledge, STDC-Net is the first framework to integrate SHAP-based feature attribution within a dual-channel CNN-GAT architecture for HD-sEMG gesture recognition, thereby balancing high accuracy with enhanced model transparency and performance.

## 2. Method

**Figure 1:**
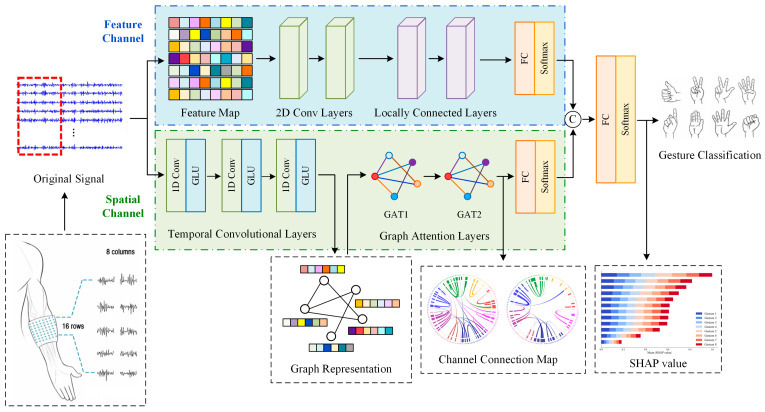
The overall architecture of STDC-Net.

HD-sEMG signals contain rich information across the time, frequency, and spatial domains. STDC-Net is constructed to incorporate features from these three domains to enhance understanding of the degree of attention the network allocates to these different features. The overall architecture of STDC-Net is illustrated in Figure 1. The Feature Channel focuses on time-domain and frequency-domain features extracted from HD-sEMG signals to facilitate fast network convergence. SHAP values are employed to rank feature contributions, with larger values indicating higher priority (i.e., greater importance in classification). The Spatial Channel focuses on temporal-domain and spatial-domain features by combining temporal convolutional layers with spatial graph attention layers. The input to the Spatial Channel is the original HD-sEMG signal. Temporal features are extracted using 1D convolutional layers followed by the gated linear unit (GLU) [31] to introduce nonlinearity and compensate for any incomplete information in the Feature Channel. Moreover, the graph attention layers capture the spatial-domain features, followed by the channel connection maps to understand the synergy between muscles.

### 2.1. Feature Channel with Convolutional Layers

In order to understand the degree of attention paid by the network to different features during gesture recognition, the features widely used for EMG signal classification are extracted and ranked, including 15 time-domain and 6 frequency-domain features. By incorporating all the features that have been previously applied in similar tasks [32,33,34], it helps to better understand which features play a more significant role in improving classification performance.

**Figure 2:**
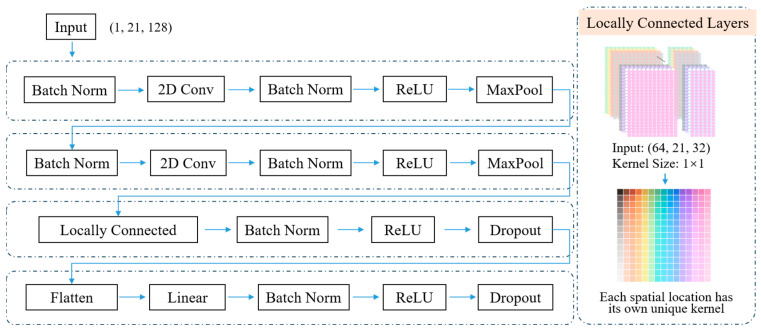
The structure of the Feature Channel.

2D convolutional layers process information across the feature dimension and channel dimension to obtain high-dimensional feature representations. Locally connected layers further refine the detailed feature extraction. The convolution weights at different spatial positions are not shared, allowing the network to learn multi-scale features and improving its ability to recognize specific local patterns. The detailed structure of the Feature Channel is shown in Figure 2.

The Feature Channel takes a 2D input of size 21 (handcrafted features) × 128 (EMG channels). It comprises two standard 2D convolutional blocks (3 × 3 kernels, 16 → 64 filters) with batch normalization, ReLU, and max-pooling, followed by two locally connected blocks (64 filters, dropout 0.5) to capture position-specific patterns. A final fully connected layer compresses the feature map to a 64-dimensional embedding.

The 15 time-domain features include: waveform length (WL), slope sign change (SSC), root mean square (RMS), kurtosis (KU), Willison amplitude (WAMP), zero crossing (ZC), median, mean, max, min, peak to peak (PK), average rectified value (ARV), variance (VA), standard deviation (ST) and skewness (SK). The 6 frequency-domain features include: total power, mean frequency (Mean_freq), median frequency (Median_freq), peak frequency (Peak_freq), average power (Avg_power) and spectral entropy value (Entropy_val).

After classification, SHAP values are used to evaluate the contribution of each feature. SHAP is a widely used feature-attribution approach in explainable AI, derived from cooperative game theory (CGT) to explain prediction results, especially in black-box models [35]. SHAP quantifies the contribution of each feature and explains the model’s prediction as the sum of the Shapley values of each input feature.

### 2.2. Spatial Channel with Graph Attention Layers

**Figure 3:**
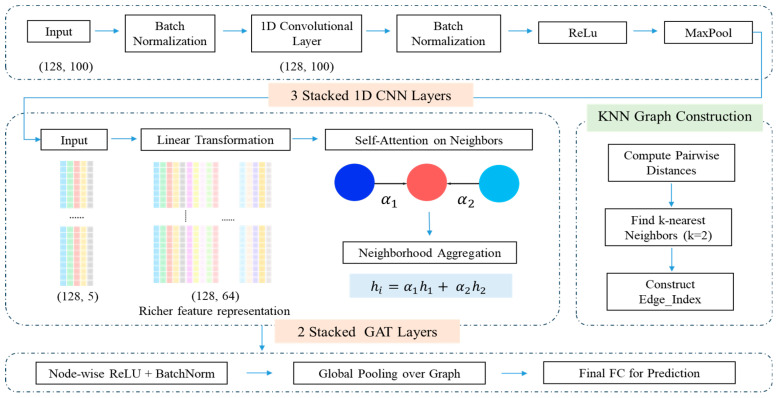
The structure of the Spatial Channel.

The Spatial Channel consists of temporal convolutional layers and the spatial graph attention layers, as shown in Figure 3. Temporal convolutional layers, constructed by 1D convolutional layers and GLUs, automatically extract features and complement the information that may have been omitted by the Feature Channel. While CNNs excel in handling grid-like inputs within Euclidean space, they may struggle in processing data with non-Euclidean structures such as HD-sEMG signals. To address this concern, graph attention layers are implemented to model the topological relationships within the HD-sEMG signals.

The Spatial Channel processes raw HD-sEMG signals (128 channels × 100-time samples). Temporal features are extracted via three 1D convolutional blocks (kernel size = 3, filters = 128, pool sizes 2, 2, 5), reducing the temporal dimension to 5. A k-nearest neighbor graph (k = 2) is dynamically constructed from the resulting 128 node features (each of dimension 5). Two GAT layers (single head, 5 → 64 and 64 → 64) perform spatial message passing, followed by global mean pooling and a fully connected layer to obtain a 64-dimensional embedding. The GAT outputs attention weights for generating channel connection maps.

The graph attention layer introduces the attention mechanism into the spatial domain-based graph neural network, updating node features through the representation of neighbor nodes instead of complex computations like Laplace matrices. The attention mechanism of the graph attention layer determines weights of neighboring nodes when aggregating feature information [36]. To make the coefficients comparable across nodes, the attention coefficients are usually represented as Equation (1):

(1) $$\alpha_{ij}=\mathrm{Softmax}\left(\left\{e_{ij}\right\}\right)=\frac{\exp\left(\alpha \left(WH_{i}^{k-1},WH_{j}^{k-1}\right)\right)}{\sum_{l\in N\left(i\right)}\exp\left(\alpha \left(WH_{i}^{k-1},WH_{j}^{k-1}\right)\right)}$$

where *e_ij_* represents the strength of the relationship between node *i* and node *j*; W represents the weight matrix; *H^k^*^−1^ represents the hidden node features in the (*k* − 1)th layer. Each node attends only to its neighboring nodes, with *α_ij_* interpreted as the transfer probability from node *i* to each neighboring node.

Channel connection maps are generated by the outputs of the graph attention layer, reflecting the connection weights between each channel. Through them, the interaction of muscles can be better understood.

## 3. Experimental Results

### 3.1. Datasets and Training Settings

The CapgMyo DB-a dataset [9], which is publicly available, is used to evaluate the performance of the STDC-Net model for gesture recognition. The dataset consists of HD-sEMG recordings obtained from 18 healthy able-bodied subjects (aged 23 to 26 years), each performing eight distinct hand gestures with 10 repetitions per gesture. The HD-sEMG data was recorded using eight acquisition modules, each containing a matrix-type (2 × 8) electrode array, amounting to a total of 128 channels. The inter-electrode horizontal distance was 7.5 mm, and the vertical distance was 10.05 mm.

All experiments were conducted using the PyTorch framework (version 1.13.0) on a system equipped with NVIDIA T1000 GPU (Nvidia, Santa Clara, CA, USA). The model was trained for 20 epochs with a batch size of 80. The AdamW optimizer was used to update the parameters with an initial learning rate of 0.01.

Comparative experiments and ablation experiments were conducted to evaluate the performance of the STDC-Net model. For intra-subject tasks, odd-numbered trials were separated for model training, while even-numbered trials were used for model testing. For inter-subject tasks, an 18-fold cross-validation approach was adopted, where odd-numbered and even-numbered trials per gesture were separated for model training and testing, respectively, during the fine-tuning processing stage. For fine-tuning, we used the same data split as the intra-subject task: for each target subject, odd-numbered trials were used for training (fine-tuning) and even-numbered trials for testing. The fine-tuning was performed for 10 epochs with a batch size of 20. The experimental configuration summary for all tasks is shown in Table 1.

### 3.2. Ablation Experiments

The correlation analysis and significance testing were performed on the 21 features extracted from the Feature Channel with the intra-subject task data as the training set. It should be emphasized that all feature correlation analyses, SHAP value (version 0.41.0) computations, and feature selection procedures were performed exclusively on the training set of the intra-subject task. The selected feature indices were then fixed and applied to both the training and test sets.

**Figure 4:**
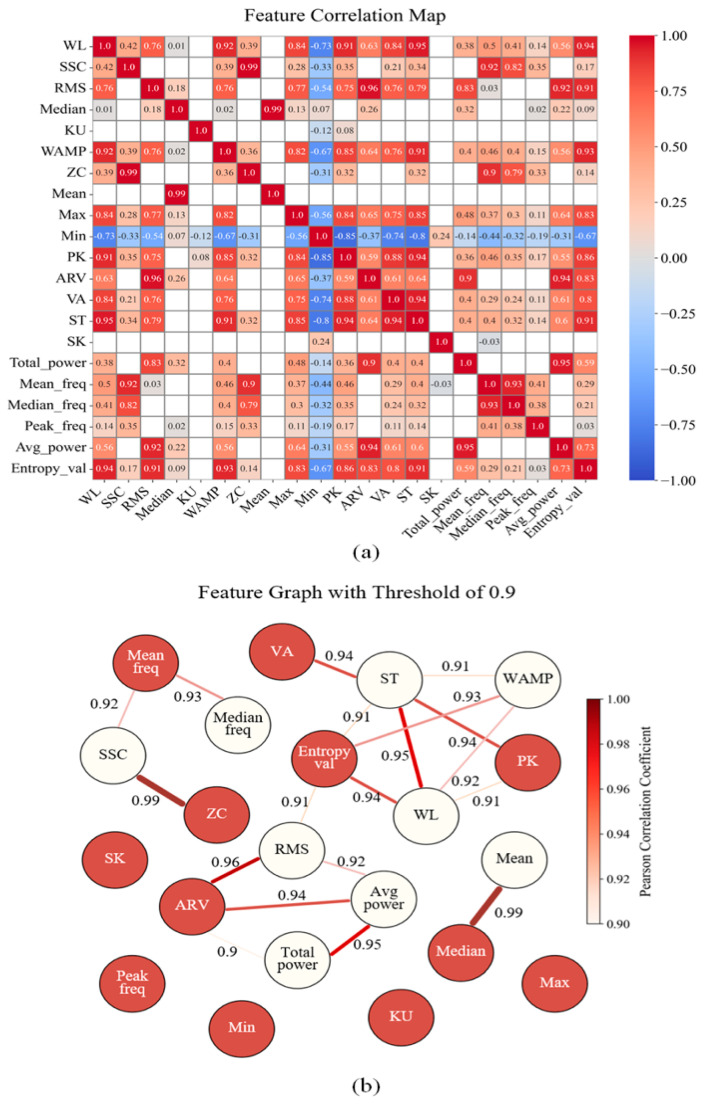
Feature correlation map. (**a**) Feature correlation map of 21 features; (**b**) Feature graph with threshold of 0.9.

To quantify the pairwise linear relationships among the 21 extracted features, Pearson correlation coefficients were computed for each gesture separately using the training set data. For each pair of features, the correlation coefficients were first calculated across all samples within a given gesture. To enable statistical testing across subjects, the Fisher Z transform was applied to convert each correlation coefficient into a Z-value, which approximately follows a normal distribution. A one-sample *t*-test was then performed on the Z-values across all subjects for each feature pair to test whether the mean correlation was significantly different from zero. To control the family-wise error rate arising from multiple comparisons, all resulting *p*-values were adjusted using the Benjamini–Hochberg false discovery rate (FDR) procedure with a significance threshold of 1%. Feature pairs with adjusted *p*-values above this threshold were considered not significantly correlated and are displayed as blank cells in Figure 4a. The heatmap of feature correlation is shown in Figure 4a, with red indicating positive correlation and blue indicating negative correlation. Blank cells indicate that there is no significant correlation between two features. Figure 4b shows the feature correlation graph between features under the significance threshold of 0.9. Nodes with no connections represent that there is no correlation between this feature and other features; it is believed that these features are independent and irreplaceable. Other features need to be filtered according to their contribution to the classification task.

To justify the choice of the correlation threshold (0.9) for feature filtering, we conducted a sensitivity analysis by varying the threshold from 0.7 to 1 in steps of 0.1. As shown in Table 2, the classification accuracy remains highly stable across all thresholds, while the number of selected features gradually decreases as the threshold increases. The computational cost also decreases accordingly. At the threshold of 0.90, the model achieves a balanced trade-off between accuracy and computational efficiency, which is why we selected this value. These results confirm that our feature selection strategy is robust and not sensitive to the exact choice of the correlation threshold.

**Figure 5:**
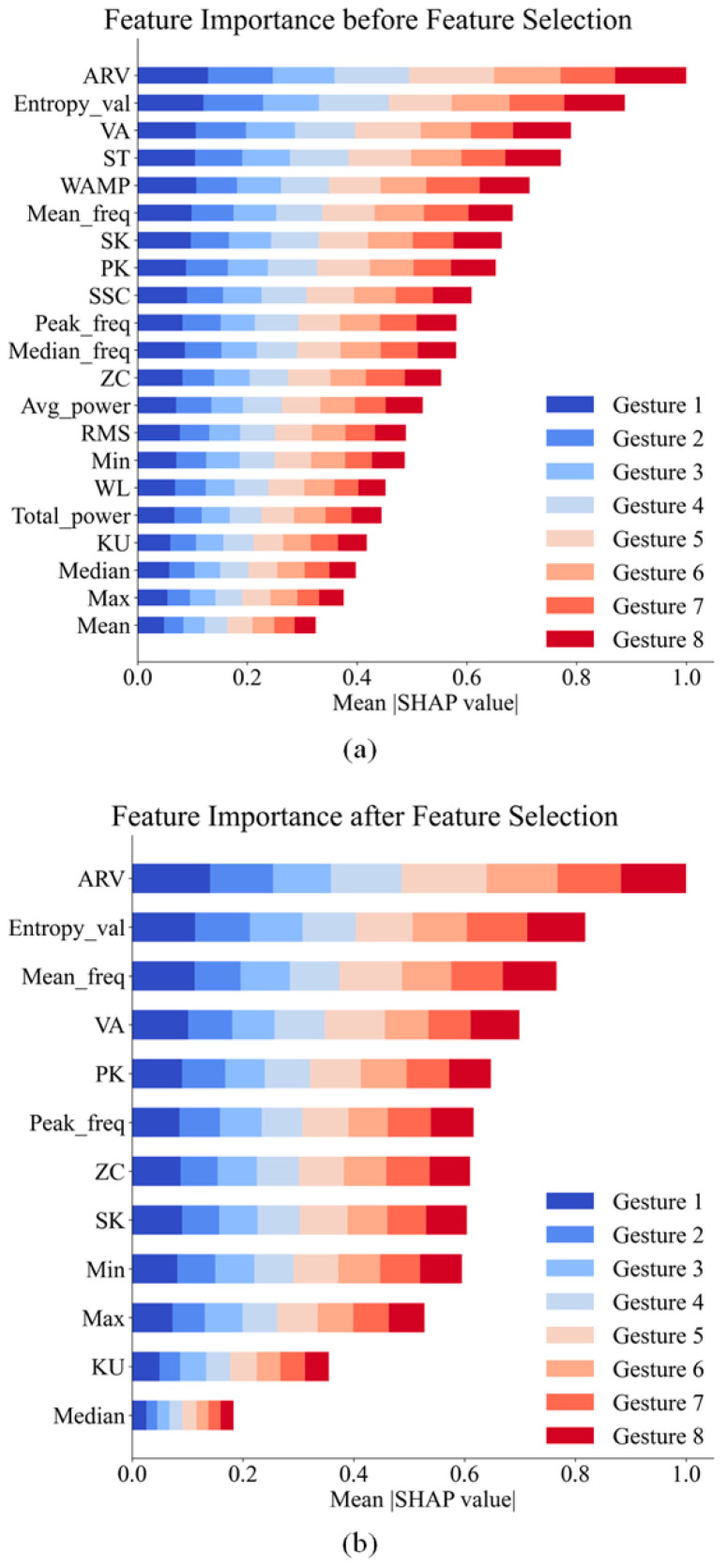
Feature importance before and after feature selection. (**a**) Feature importance before feature selection; (**b**) Feature importance after feature selection.

In order to filter features reasonably, ablation experiments on the Feature Channel were conducted. The training and testing dataset was the same as the one for the intra-subject task. Firstly, all 21 features were used, the classification results are shown in Table 2, and the feature importance map with SHAP values is shown in Figure 5a.

It should be noted that the feature importance map was generated with the training set. Based on the information from Figure 4b and Figure 5a, 12 features were selected, including: variance (VA), zero crossing (ZC), average rectified value (ARV), peak to peak (PK), median, skewness (SK), min, max, kurtosis (KU), mean frequency (Mean_freq), peak frequency (Peak_freq) and spectral entropy value (Entropy_val). The filtered 12 features were input into the Feature Channel for training, and the classification results are shown in Table 2. The feature importance after feature selection is shown in Figure 5b.

Table 2 shows that training with 12 features resulted in a 0.81% decrease in detection accuracy compared to 21 features but significantly reduced the calculating consumption with a 42.75% decrease in parameters and 42.84% decrease in Floating Point Operations (FLOPs), indicating that feature filtering helps to remove redundant features, effectively reducing model parameters and computational complexity while ensuring detection accuracy. From Figure 4, it is evident that the attention level of the model to different features is roughly the same before and after feature selection, with only a few features changed, like Mean_freq and SK. Although the features KU, median and max exhibit limited predictive power in model classification, they are preserved during the feature selection process as they demonstrate no significant collinearity with other features.

Ablation experiments were conducted to evaluate the contributions of each module in STDC-Net. Table 3 presents the recognition results for 100 ms segmentation, where the Feature Channel represents convolutional neural layers and the Spatial Channel represents graph attention layers.

Our results show that STDC-Net achieved the best performance, followed by the Feature Channel, while the Spatial Channel ranks last. The Feature Channel played a primary role as it extracted several time-domain and frequency-domain features, minimizing the feature filtering step and enabling the STDC-Net model to learn advanced feature information more efficiently. The Spatial Channel, although less dominant, adds complementary feature information that was not manually extracted from the Feature Channel.

### 3.3. Intra-Subject Gesture Recognition Results

To evaluate the performance of STDC-Net, it was compared against SOTA methods including Geng-Net [9], Hu-Net [37], Chen-Net [38], Bai-Net [39], and All-ConvNet [40].

Geng-Net is the pioneering work that introduced the concept of sEMG images with deep convolutional networks, serving as the foundational benchmark on the CapgMyo DB-a dataset. Hu-Net incorporates an attention-based hybrid CNN-RNN architecture to better capture the temporal properties of EMG signals. Chen-Net-2D/3D systematically compares 2D and 3D CNNs, where the 3D variant captures both spatial and temporal structures from sequential sEMG images. Bai-Net addresses the class-incremental learning scenario to mitigate catastrophic forgetting when new gesture classes are introduced. All-ConvNet leverages a lightweight pure-convolutional architecture with transfer learning to handle inter-session and inter-subject variability. These methods collectively cover a broad spectrum of representative approaches, from early CNN-based models to recent advances in attention mechanisms, 3D spatiotemporal modeling, class-incremental learning, and transfer learning, providing a comprehensive and fair benchmark for evaluating STDC-Net.

To comprehensively evaluate their recognition capabilities, the HD-sEMG signals were segmented into 50 ms (25 ms overlap), 100 ms (50 ms overlap) and 150 ms (75 ms overlap) before being fed into the models. Basic information and recognition results are summarized in Table 4.

As shown in Table 4, STDC-Net outperforms all other SOTA methods across all segmentation windows. Notably, in the case of 150 ms segmentation, STDC-Net reached an accuracy of 99.8%. Compared to the suboptimal Chen-Net-2D method, STDC-Net handled signals of various lengths perfectly, demonstrating superior robustness. In addition, we measured the inference throughput of STDC-Net on an NVIDIA T1000 GPU; the model achieves an average inference speed over 1000 FPS, which is far beyond the typical real-time requirement of 30 FPS for prosthetic control applications [41]. This confirms that STDC-Net not only achieves high accuracy but also satisfies practical real-time deployment requirements.

### 3.4. Inter-Subject Gesture Recognition Results

While STDC-Net performs well in the intra-subject recognition task, real-world applications typically require inter-subject generalization. To evaluate this, an 18-fold cross-validation approach was implemented, and the recognition results are shown in Table 5.

It can be seen from Table 5 that the performance of the Feature Channel, Spatial Channel and STDC-Net on the inter-subject task shows a significant decline compared to the intra-subject task, with a decrease in accuracy of 60.98–64.03%, reflecting the differences in HD-sEMG signals among the subjects. Meanwhile, the recognition accuracy after fine-tuning shows a significant improvement of 150.21–169.17%, demonstrating the substantial impact of fine-tuning on the STDC-Net model. The results in Table 5 follow the same trends of Table 4 in that STDC-Net performed the best, followed by the Feature Channel and finally the Spatial Channel.

**Figure 6:**
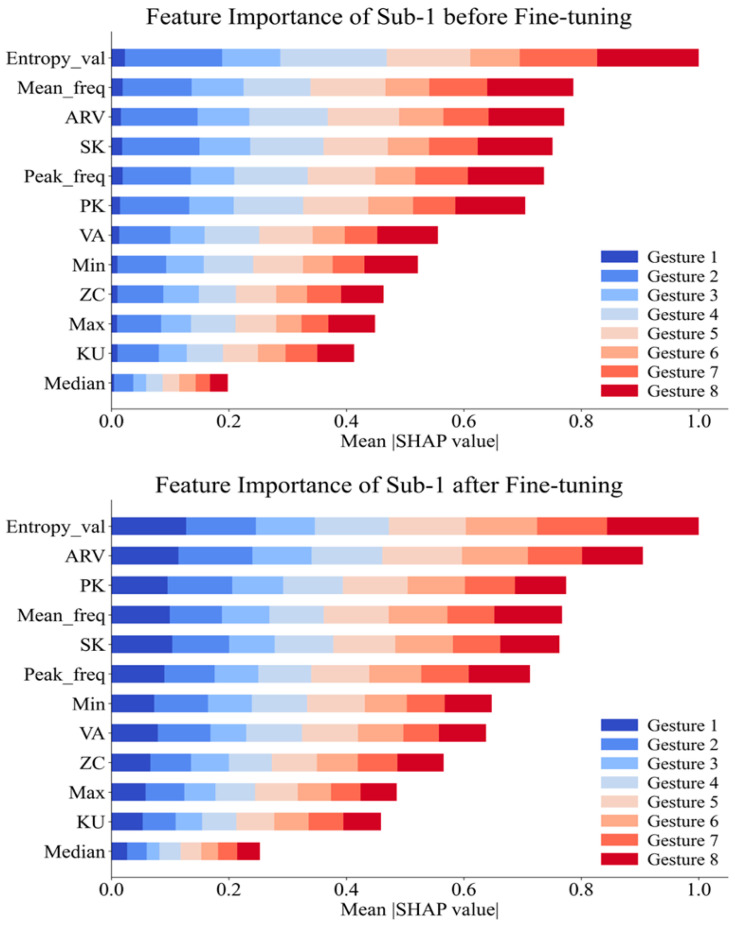
Feature importance of Subject 1 before and after fine-tuning.

To visually demonstrate the changes in weights before and after fine-tuning, SHAP values and channel connection maps are used to visually analyze the outputs of the Feature Channel and Spatial Channel. Taking Subject 1 as an example, the feature importance maps before and after fine-tuning are shown in Figure 6.

It can be seen that the attention level of the model to different features is roughly the same before and after fine-tuning, with model-adjusted weights of a few specific features, allowing the fully connected layer to learn new information. Moreover, it can be observed that between different subjects, the features focused on by STDC-Net vary both before and after fine-tuning, highlighting the differences among various subjects. To gain a more comprehensive understanding, we conducted further investigation in comparison with Figure 5b. Quantitative analysis revealed that feature importance rankings demonstrate cross-subject stability, despite observable fluctuations in absolute contribution magnitudes. As evidenced in Figure 5b, the top-tier features (ARV, Entropy_val, Mean_freq) retained their prominence across individual subject analyses, while the lowest-ranked features (Max, KU, Median) persistently occupied the lowest tiers, reinforcing the cross-subject stability of hierarchical feature importance.

**Figure 7:**
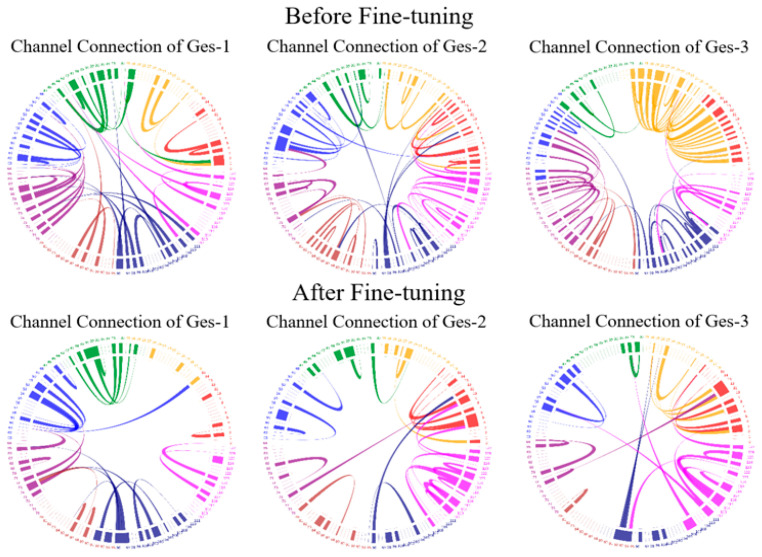
Channel connection of gestures 1–4 in Subject 1 before and after fine-tuning.

In the Spatial Channel, the GAT module updates the edge index and edge weights during training, enabling the generation of channel connection maps for a clearer visualization of the relationship between channels with respect to each gesture. Taking gestures 1–3 of Subject 1 as an example, the corresponding channel connection maps are provided in Figure 7.

To better illustrate the interaction between each channel, only the top 1% of edge weights are shown. The lines inside the circle represent the connection between channels, and the size of the rectangles on the outer circle represents the weight contribution of each channel. It is noted that different colors in Figure 7 mean different electrodes. Red represents electrodes 0–15, yellow represents electrodes 16–31, green represents electrodes 32–47, blue represents electrodes 48–63, purple represents electrodes 64–79, brown represents electrodes 80–95, dark blue represents electrodes 96–111, and pink represents electrodes 112–127. As shown in Figure 7, before fine-tuning, the connection strengths are relatively uniform across channels. After fine-tuning, the network exhibits a sparser set of connections, indicating that it learns to identify and focus on the most relevant muscle groups for specific gestures more accurately.

## 4. Discussion

Gesture recognition for daily living has attracted much attention for its impact on health care and human–computer interaction. The dual-channel structure of STDC-Net fully considers both feature and spatial factors contained in HD-sEMG signals, achieving SOTA results on intra-sub recognition tasks. Moreover, STDC-Net achieves excellent results on inter-sub recognition tasks after fine-tuning, effectively reducing inter-sub differentiation, which facilitates building real-time analysis models.

In addition, STDC-Net demonstrates good interpretability, as it can elucidate the feature and spatial information in the HD-sEMG signals. Regarding feature information, Figure 5 shows the feature importance ranking contributing to classification, and Figure 6 presents the feature importance ranking before and after fine-tuning for the inter-sub tasks. Both figures share similarities in that the average rectified value (ARV) and spectral entropy value (Entropy_val) are the two most important features for classification, suggesting that these two features can capture the differences in HD-sEMG signals across different gestures. In contrast, max, kurtosis (KU), and median consistently rank lowest, indicating their relatively little contribution to gesture recognition via HD-sEMG signals. The distribution of ARV, Entropy_val, max, KU and median demonstrates inter-sub homogeneity, while the importance of other features varies among subjects, reflecting the specificity between each subject. Furthermore, Figure 6 reveals that fine-tuning alters the model’s preference for different features, demonstrating that the process essentially modifies the weight distribution of features in the network. Regarding spatial information, Figure 7 shows the channel connection maps before and after fine-tuning, reflecting that fine-tuning enables the network to more accurately identify and focus on muscle groups most relevant to specific gestures. Additionally, the distinct channel connection maps observed across different gestures confirm that distinct muscle groups are activated for different gestures.

Although STDC-Net achieves SOTA results on recognition tasks and provides explanations linking features and gestures, there are still some limitations. It should be noted that, as the CapgMyo DB-a dataset does not provide electrode-to-muscle anatomical annotations, the channel connection maps presented in this study reflect electrode-level functional coupling rather than direct physiological muscle synergy. Future work using datasets with precise muscle-level annotations or imaging-guided electrode placement will be required to validate the physiological underpinnings of these spatial connectivity patterns. It should be noted that the interpretability analysis in this study is primarily qualitative and post hoc in nature. Quantitative evaluation of explanation quality remains an open challenge in the XAI community without established standards, and we leave this as future work. In addition, subject variability results in relatively low recognition performance for inter-subject tasks, although fine-tuning reduces the inter-subject differences and improves the recognition accuracy. Future studies could implement data augmentation or transfer learning methods to enhance generalization through data-driven or model-based approaches.

Another limitation of this study is that all experiments were conducted on a single public dataset (CapgMyo DB-a). While we performed intra-subject, inter-subject, and fine-tuning evaluations to comprehensively assess model performance from multiple angles, we acknowledge that validation on additional datasets would further strengthen the evidence for generalizability. Future work will extend our evaluation to other public HD-sEMG datasets, such as the CSL-HDEMG dataset or NinaPro, to verify the cross-dataset robustness of STDC-Net.

## 5. Conclusions

This study introduced STDC-Net to address the challenges of interpretability and network optimization commonly encountered by current deep learning-based gesture recognition. The dual-channel structure of STDC-Net allowed for automatic extraction of relevant temporal, spectral, and spatial biomarkers from HD-sEMG signals, decomposing the network attention mechanism from different perspectives, including the time domain, frequency domain and spatial domain. STDC-Net outperformed other state-of-the-art methods in gesture recognition, as validated on the comprehensive CapgMyo DB-a dataset. The model strikes a balance between performance and interpretability, providing insights into its decision-making process while ensuring high accuracy. These findings emphasize the value of integrating interpretability and optimization into deep learning frameworks to advance the practicality and reliability of myoelectric control systems. Future research should focus on transfer learning approaches, such as domain adaption and domain generalization, to eliminate individual difference and to improve the generalizability across diverse users. Additionally, an interesting future direction is to investigate whether the convergence process during training can guide further structural optimization of the dual-channel architecture, potentially leading to innovative performance gains beyond the current fixed design.

## Figures and Tables

**Table 1:** Experimental configuration summary for all tasks.

Task	Data Split	Epoch	Batch Size	Length
Intra-sub	Odd trials (train)/Even trials (test), per subject	20	80	50/100/150 ms
Inter-sub (w/o FT)	Leave-one-subject-out (18-fold), per fold	20	80	100 ms
Inter-sub (with FT)	Same 18-fold, per target subject: odd trials (FT)/even trials (test)	10	20	100 ms

**Table 2:** Ablation study results on feature selection.

Threshold	Feature Number	Params (M)	FLOPs (M)	Accuracy (%)	Precision (%)	Recall (%)	F1 (%)
0.7	10	1.32	7.85	95.71	95.73	95.71	95.71
0.8	10	1.32	7.85	95.71	95.73	95.71	95.71
0.9	12	1.58	9.42	96.55	96.57	96.55	96.55
1	21	2.76	16.48	97.34	97.35	97.34	97.34

**Table 3:** Ablation study results on CapgMyo DB-a dataset.

Method	Accuracy (%)	Precision (%)	Recall (%)	F1 (%)
Feature Channel	96.55	96.57	96.55	96.55
Spatial Channel	89.84	90.12	89.43	89.44
STDC-Net	99.70	99.71	99.69	99.69

**Table 4:** Basic information and recognition results of SOTA methods on CapgMyo DB-a dataset.

Method	Year	Accuracy (%)		
		50 ms	100 ms	150 ms
Geng-Net	2016	99.0	–	99.5
Hu-Net	2018	99.3	–	99.7
Chen-Net-2D	2020	95.3	99.6	97.1
Chen-Net-3D	2020	96.7	98.3	98.6
Bai-Net	2024	–	–	94.0
All-ConvNet	2024	96.8	97.4	98.0
STDC-Net	–	99.5 ± 0.85	99.7 ± 0.44	99.8 ± 0.32

**Table 5:** Inter-subject recognition results.

**Subject**	**Before Fine-Tuning**		
	**Accuracy (%)**		
	**Feature Channel**	**Spatial Channel**	**STDC-Net**
1	18.55	20.30	23.42
2	64.21	44.67	73.03
3	33.95	28.55	36.71
4	42.24	33.09	51.05
5	59.61	30.65	63.42
6	25.26	29.28	28.16
7	38.95	55.72	42.50
8	30.66	27.83	28.55
9	37.11	36.31	46.18
10	14.74	20.07	18.55
11	10.92	9.87	15.66
12	23.03	28.36	25.26
13	37.89	54.74	36.58
14	11.97	22.63	17.39
15	49.34	29.80	52.24
16	57.50	39.28	62.37
17	44.87	34.01	50.26
18	44.34	36.45	48.82
Average	35.84 ± 15.76	32.31 ± 11.18	38.90 ± 16.69
**Subject**	**After Fine-Tuning**		
	**Accuracy (%)**		
	**Feature Channel**	**Spatial Channel**	**STDC-Net**
1	96.84	79.08	97.24
2	97.63	89.21	97.76
3	98.95	85.66	98.95
4	99.87	81.45	99.87
5	90.13	86.32	91.84
6	99.87	89.74	100.00
7	97.24	80.00	99.21
8	97.50	86.45	99.47
9	98.55	90.39	98.68
10	91.84	88.55	93.29
11	94.87	74.87	95.00
12	94.08	85.53	96.18
13	98.95	92.24	98.55
14	95.92	74.34	97.63
15	96.32	84.21	97.76
16	91.58	79.08	94.74
17	98.68	83.55	99.61
18	97.63	88.16	97.89
Average	96.47 ± 2.82	84.38 ± 5.10	97.33 ± 2.53

## Data Availability

The data presented in this study are available on request from the corresponding author.
